# Numerical Analysis of Fluid Flow and Heat Transfer in Micro-Channel Heat Sinks with Double-Layered Complex Structure

**DOI:** 10.3390/mi11020146

**Published:** 2020-01-29

**Authors:** Xiaogang Liu, Meng Zhang, Zhongyi Wang, Juhui Chen, Haiou Sun, Haifeng Sun

**Affiliations:** 1 School of Mechanical and Power Engineering, Harbin University of Science and Technology, Harbin 150001, China; xiaogang_liu@hrbust.edu.cn (X.L.); chenjuhui@hit.edu.cn (J.C.); sunhaifeng2210@163.com (H.S.); 2 College of Power and Energy Engineering, Harbin Engineering University, Harbin 150001, China; sunhaiou@hrbu.edu.cn; 3 School of Energy Science and Engineering, Harbin Institute of Technology, Harbin 150001, China

**Keywords:** microchannel heat sink, heat transfer enhancement, secondary flow

## Abstract

Micro-channel heat sink (MCHS) has been extensively used in various electronic cooling fields. Double-layered MCHS, or DL-MCHS, is regarded as one effective technique for high-heat-flux transfer and is expected to meet the ever-increasing heat load requirement of future electronic device generations. In order to improve the cooling capacity, two new types of the MCHS, with a double-layered matrix structure (DL-M) and double-layered interlinked matrix structure (DL-IM) are proposed and investigated numerically. The two designs are compared with the traditional double-layered rectangular structure (DL-R) and the double-layered triangular structure (DL-T). Different properties of the heat sink are investigated to assess the overall heat transfer performance, for which coolant flow and heat transfer are both evaluated. The numerical results reveal that the periodical slot subchannel in the matrix has a significant effect on fluid flow for heat transfer. In comparison to the DL-R and the DL-T, the DL-M and DL-IM realize a much lower pressure drop and temperature rise at the base surface and also have higher Nusselt number and secondary flow intensity, therefore, manifesting better overall thermal performance. In the DL-M and DL-IM, the coolant flows along the periodical subchannel in one layer and is redirected into the second layer with vortices being induced. The vortices promote the coolant mixing and enhance the mass and heat transfer. These geometric design strategies can provide references for wide heat sink applications.

## 1. Introduction 

With the rapid development of micro-electro-mechanical systems (MEMSs) in the past decades, the heat generation in many compact electronic devices can easily exceed 10^6^ W/m^2^ [1]. The heat is transferred through heat exchangers, which is usually in a relatively large scale. Compared with the traditional heat exchanger, the microchannel heat sink (MCHS) with miniaturized size can greatly increase the coolant contact surface area and hence reduce the device scale. Therefore, MCHS is regarded as one of the effective techniques for high-heat-flux removal and has become a hot research focus in recent years.

The single-layered microchannel heat sink (SL-MCHS) has been extensively used in various electronic devices for cooling purposes. Later, to meet the fast growing miniaturization and integration trend of electronic devices, a double-layered microchannel heat sink (DL-MCHS) was proposed. Vafai et al. firstly designed a MCHS with a double-layered rectangular structure [2]. They pointed out that both temperature rise and pressure drop in the device can be lowered compared to that in the SL-MCHS.

DL-MCHS with a rectangular cross-section structure was later intensively studied by many researchers. Wei et al. [3] numerically investigated the double-layered rectangular microchannel structure with a coolant in parallel flow arrangement. They found the required pumping power and flow rate in this condition was lower than that for SL-MCHS. Then, Wei et al. [4] performed experimental and numerical work on the effects of coolant flow direction and the flow rate allocation among layers. It is demonstrated that the counter-flow arrangement provides better temperature uniformity over the tested flow rate, and excellent overall cooling performance was obtained by the stacked microchannel heat sink in their experiment. Levac et al. [5] discussed the effects of Reynolds number, flow arrangement, and inlet velocity profile on the overall thermal resistance, peak temperature and pumping power on the heated surface of SL-MCHS and DL-MCHS. At low Reynolds number condition, they found that the thermal resistance of heat sink with parallel-flow was lower than that with counter-flow, whereas counter-flow arrangement showed a more uniform bottom temperature. Wu et al. [6] numerically investigated the DL-MCHS with different aspect ratios and width ratios in different flow conditions. They found at a given pumping power, the overall performance of DL-MCHS can be improved by adjusting the inlet velocity of upper channels to being slower than that of bottom channels. In addition to the above investigation, Radwan et al [7], Ling et al [8] and Zuo et al [9] conducted a series of further optimized works by using different numerical or experimental approaches.

Compared to the simple rectangular cross-section microchannel, DL-MCHS with a complex structure is more effective in heat transfer. The complex structure can be grooves, ribs, dimples, cavities or ribs aside the rectangular microchannel wall, which provides a higher flow area to volume ratio, induces the secondary flow, disturbs the thermal and hydrodynamic boundary layers, and thus enhances the heat transfer [10]. The DL-MCHS with trapezoidal structure was proposed by Sharma et al. [11], which manifests better thermal resistance and temperature variations performance over the traditional MCHS. Xie et al. [12] studied a DL-MCHS with a wavy structure, which showed to be superior in the aspects of thermal performance compared to the simple structure DL-MCHS. Zhai et al. [13] first proposed a novel DL-MCHS type with triangular rips and triangular cavities, which realizes more uniform temperature distribution on the bottom wall than that in simple structured MCHSs. In addition, DL-MCHS with a complex structure can effectively eliminate the internal thermal stress in microelectronic equipment. Then, Zhai et al [14] researched three other kinds of DL-MCHS with triangular rips and triangular cavities considering the field synergy principle and secondary flow characters. They concluded that the arrangement of cavities and ribs has a great impact on flow friction and heat transfer and pointed out there is a large potential to enhance the convective heat transfer in double-layered microchannels. Pouzesh et al. [15] studied MCHSs with Y-shaped cavities, which was found to have high reliability and effectiveness in the performance. In addition, based on the constructal theory, Hajmohammadi et al. [16,17], Li et al. [18] and Zhang et al. [19] separately proposed the square-shaped and T-Y shaped assembly of fins, MCHSs with Y-shaped bifurcation plates and MCHSs with multiple bifurcations for the heat removal purpose.

One important reason that many studies focused on the DL-MCHS with complex structure can be attributed to the secondary flow produced in the complex structure. The secondary flow is normal to the main flow and easily formed in the cavities or grooves zones. Literatures [20,21,22,23] have researched the effect of secondary flow on the thermal performance in both single-layered and double-layered microchannels, and they found that the secondary flow has a positive effect to enhance the heat transfer in the MCHSs. 

Under the condition of a constant heating surface area and heat dissipation power, the purpose of the MCHS design is to obtain excellent overall thermal performance. Thus, to consider the coolant flow and heat transfer systematically, the maximum and average temperature on the base surface, thermal resistance, pumping power, Nusselt number, thermal-enhanced factor, and secondary flow intensity are investigated as the criteria to evaluate the heat transferred performance. In this paper, we designed two novel double-layered matrix microchannels and evaluated the MCHS by the above-mentioned criteria.

## 2. Model Design

### 2.1. Physical Model

**Figure 1:**
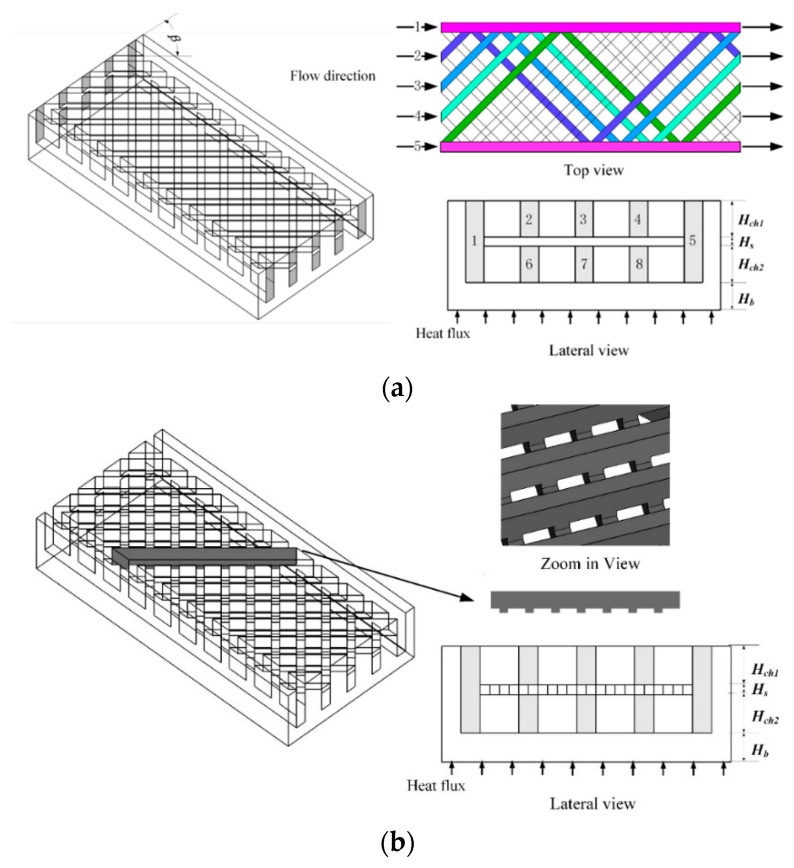

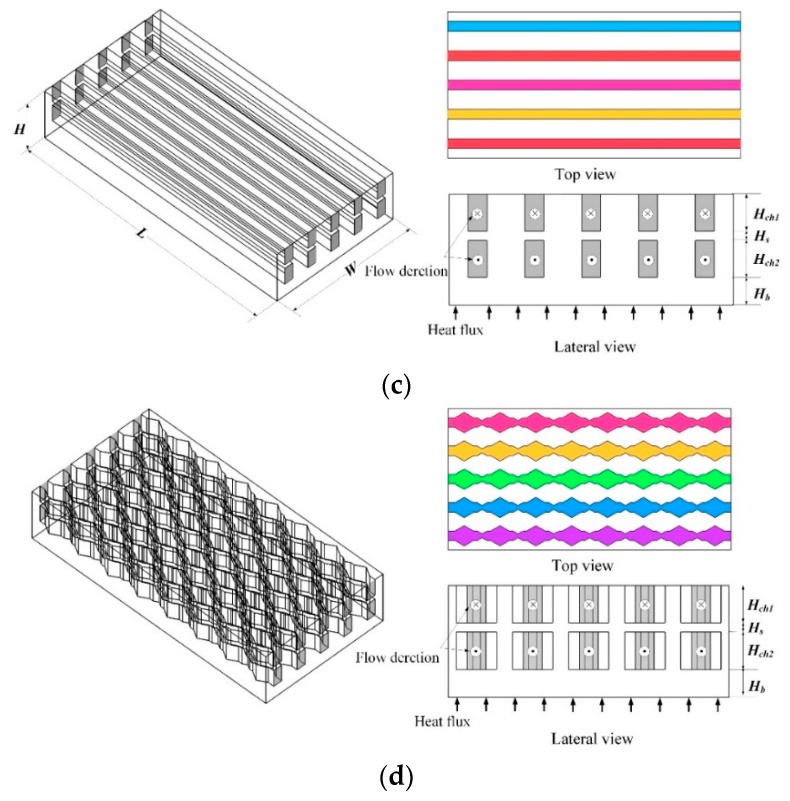
Schematic of four double-layered micro heat sinks: (**a**) double-layered matrix (DL-M) microchannel, (**b**) double-layered interlinked-matrix (DL-IM) microchannels, (**c**) double-layered microchannel with rectangular cross-section (DL-R), and (**d**) double-layered microchannel with triangular ribs and triangular cavities (DL-T).

The conventional microchannel MCHS usually has very complex configuration, such as pin fin, variable pin fin, curved channels, and a tree-like or biomimetic structure, which prevents massive fabrication of the device. In addition, the complex structure usually results in high flow resistance, high thermal stress and low coolant flow uniformity. In this paper, inspired by the trailing-edge cooling system for gas turbines [24,25], we proposed two MCHS structures with relatively simple configurations, namely the double-layered matrix (DL-M) and the double-layered interlinked-matrix (DL-IM) as shown in Figure 1a and b. In DL-M, it consists of double layers with eight inlets. As can be seen in the lateral view of DL-M in Figure 1a, inlets 2, 3 and 4 are in the top layer; inlets 6, 7 and 8 are in the bottom layer; and inlets 1 and 5 connect both layers. The channels from inlet 2, 3 and 4 and from 6, 7 and 8 will extend to the other layer at their turning points where they interconnect with the slot channels from inlet 1 and 5, which are illustrated with the pink color. As a result, it is possible for the coolant to flow across different layers in the DL-M. The structure of DL-IM is the same with that of DL-M, except that the channels on the top layer and bottom layer are also interconnected at their top-view cross points, as illustrated in Figure 1b. The coolant is then able to flow across different layers and channels.

The two structures are designed in straight channels that can be easily fabricated using the wire-electrode cutting method. A reduced flow resistance and enhanced coolant mixing can also be realized due to the multiple interconnections between channels. We also compared their performance with that of two regular configurations, which are a double-layered microchannel with a rectangular cross-section (DL-R) [2] and a double-layered microchannel with triangular ribs and triangular cavities (DL-T) [23] as shown in Figure 1c,d, respectively. DL-R has a double layer with the same rectangular channel structure on each layer. The coolant flows from left to right in the top layer, while from right to left in the bottom layer, making it a counter-flow condition in both layers. DL-T has the same structure and flow condition with that of DL-R, except that the channel is in a triangular rib and triangular cavity shape as illustrated in Figure 1d. For all above four structures, a constant heat flux is applied to the bottom wall of the heat sink to investigate and compare their cooling performance. The nomenclature used in this paper is list in Table 1 and the details of the dimensions of the four micro heat sinks are listed in Table 2.

### 2.2. Mathematical Model

The numerical analysis is conducted by the commercial software ANSYS FLUENT 19.2 (ANSYS Inc, Pittsburgh, PA, USA) based on the finite volume method. A three-dimensional conjugate heat transfer model is used to evaluate the heat transfer between the micro heat sink material and the coolant. Deionized water is used as the coolant, which is in steady and laminar flow by controlling the Reynolds number below 350. The governing equations for the continuity, momentum and energy conservation are listed in Equations (1)–(3), respectively:

(1) $$\frac{\partial }{\partial x_{i}}(\rho u_{i})=0$$

(2) $$\frac{\partial }{\partial x_{i}}(\rho_{f}u_{i}u_{j})=-\frac{\partial p}{\partial x_{j}}+\frac{\partial }{\partial x_{i}}\left[\mu_{f}\left(\frac{\partial u_{j}}{\partial x_{i}}+\frac{\partial u_{i}}{\partial x_{j}}\right)\right],i,j=1,2,3$$

(3) $$\frac{\partial }{\partial x_{i}}(\rho_{f}u_{i}c_{pf}T)=\frac{\partial }{\partial x_{i}}\left(\lambda_{f}\frac{\partial T}{\partial x_{i}}\right)+\mu_{f}\left[2{\left(\frac{\partial u_{i}}{\partial x_{i}}\right)}^{2}+{\left(\frac{\partial u_{j}}{\partial x_{i}}+\frac{\partial u_{i}}{\partial x_{i}}\right)}^{2}\right]$$

The energy conservation equation in the solid substrate can be expressed as

(4) $$\frac{\partial }{\partial x_{i}}\left(\lambda_{s}\frac{\partial T}{\partial x_{i}}\right)=0$$

Here the transient term in Equation (4) is neglected as we applied the constant inflow condition and bottom wall heat flux; the micro heat sinks conjugate heat transfer is considered as steady. The substrate material of all cases is set to be silicon, its thermophysical properties are the same with that in Wu et al.’s research [6]. The temperature fields in both fluid and solid regions are then obtained simultaneously by conjugate computation of the above equation.

The deionized water properties in the microchannel are shown in Equations (5) and (6); the temperature-dependent thermal conductivity and dynamic viscosity are integrated into the fluid governing equations.

(5) $$\lambda_{f}(T)=-1.079257+9.43573\times 10^{-3}T-1.266071\times 10^{-5}T^{2}$$

(6) $$\mu_{f}(T)=2.414\times 10^{-5}\times 10^{\frac{247.8}{T-140}}$$

Uniform inflow rate and temperature (293 K) are set for the coolant at the inlet, with the corresponding Reynolds number ranging from 60 to 350, while the ambient pressure is applied at the outlet. A constant heat flux of 1 × 10^6^ W/m^2^ is applied to the bottom wall of the heat sink with all other walls in an adiabatic boundary condition. Continuity of temperature and heat flux are set at the solid–liquid interface. Pressure and velocity coupling are implemented by Semi-Implicit Method for Pressure-Linked Equation Consistent (SIMPLEC) algorithm. The convective term is discretized by second order upwind scheme, while the diffusion term uses the QUICK discretization scheme. In the solid region, only the energy equation is solved. The convergence criterion is set with the maximum residual less than 10^−6^, and the separated solver is used for the low speed flow.

**Figure 2:**
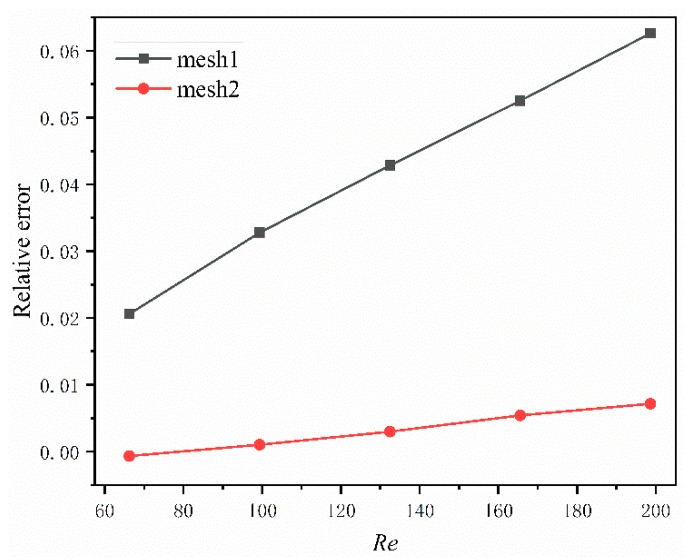
Grid-independent test for DL-T micromachine structure.

**Figure 3:**
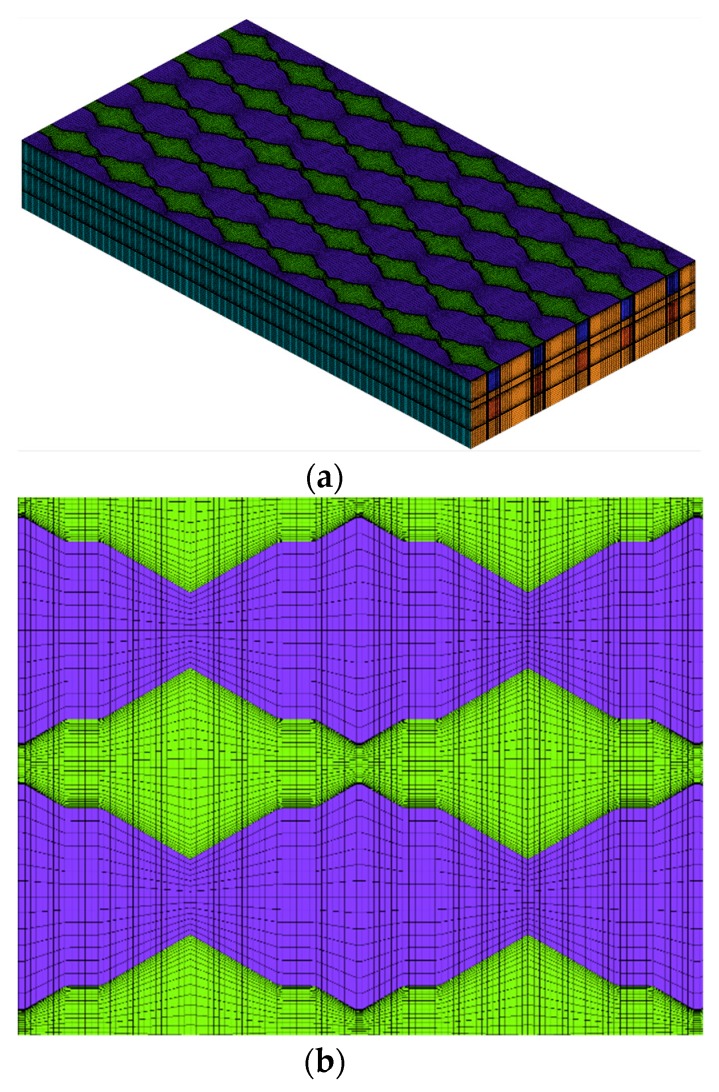
(**a**) Global mesh and (**b**) detail mesh of DL-T.

To improve the accuracy of the numerical results, the grid independence test is done firstly to ensure numerical simulation reliability. Figure 2 illustrates the grid dependence by evaluating the pressure drop in DL-T with a hexahedral mesh, which is finer in the fluid and coarser in the solid. The pressure drop ∆*P* of three different grid numbers, mesh1 (2.1 million), mesh2 (8.0 million) and mesh3 (10 million) are numerically calculated. The relative error, defined as (∆*P*_3_*−∆P_i_*)/∆*P*_3_, (*i* = 1, 2), are plotted in Figure 2 at different Reynolds numbers, where ∆*P_i_* is the pressure drop for mesh *i*. As can be seen, the sensitivity of the pressure drop is decreasing as the grid numbers increase, the maximum relative error is only 0.7% in the grid number of mesh2, which is thus chosen for further simulation work. Figure 3 demonstrates the computation mesh; the hexahedron meshes are generated for the entire computational domain by software ICEM CFD 19.2 (ANSYS Inc, Pittsburgh, PA, USA); near wall regions are made dense to capture the fluid flow and heat transfer characters.

### 2.3. Data Processing

For laminar flow, Bejan [26] developed a theory correlation for the friction factor in the rectangular microchannel, which can be written as

(7) fRe=24(Hch2+Wch2)(Hch+Wch)2

The average friction factor in the simulation can be calculated by

(8) $$f=\frac{\Delta pD_{h}}{\rho_{f}L_{ch}u_{m}^{2}}$$

where ∆*p* is the average pressure drop among the first and second layer of the microchannel, *L_ch_* is the microchannel length, and *u_m_* is the average velocity of the coolant. *D_h_* denotes the hydrodynamic diameter on the inlet and is determined as *D_h_* = 4*A*_cross_/*L*_perimeter*,*_ where *A*_cross_ is the cross area and *L*_perimeter_ is the perimeter of the channel.

The heat transfer coefficient and Nusselt number are defined as

(9) $$Q=\dot{m}c_{pf}(T_{out,f}-T_{in,f})$$

(10) $$h=\frac{Q}{A_{ch}(T_{b}-T_{f})}$$

(11) $$Nu=\frac{hD_{h}}{\lambda_{f}}$$

where $\dot{m}$ represents the mass flow rate, *Q* denotes the heat input calculated by the simulation, and *Nu* is the Nusselt number representing the heat transfer enhancement in a fluid layer as a result of convection relative to conduction across the same fluid layer. *T_out,f_* and *T_in,f_* are the outlet and inlet average temperature; *T_f_* = *(T_out,f_* + *T_in,f_* )/2 is the average coolant temperature; and *A_ch_* is the total contact area between the solid and fluid. 

The expression of local intensity of secondary flow *Se_s_*(*x*) and volume average *Se* can be written as

(12) $$Se_{s}(x)=\frac{\rho_{f}D_{h}^{2}}{\mu_{f}}\iint_{A(x)}\left|\omega^{n}\right|dA/\iint_{A(x)}dA$$

(13) $$Se=\frac{\rho_{f}D_{h}^{2}}{\mu_{f}}{∭}_{\Omega }\left|\omega^{n}\right|dV/{∭}_{\Omega }dV$$

where $\omega =\nabla \cdot \vec{v}$ denotes the vorticity, and *ω^n^* is the component of *ω* normal to the cross section. The Reynolds number is determined by

(14) Re=ρfumDhμf

The thermal resistance of micro heat sink, *R_th_* is defined as in Wu et al.’s research [6]

(15) $$R_{th}=\frac{T_{w,\max}-T_{in}}{Q}$$

where *T_w,max_* is maximum wall temperature of the bottom heated surface, and *T_in_* is the inlet temperature of the coolant. Pumping power of the heat sink, *PP* is defined as,

(16) $$PP=\sum_{i}^{n}\dot{v}\Delta p_{i}$$

where $\dot{v}$ and ∆*p* represent the volume flow rate and pressure drop, respectively in each layer.

To evaluate the overall flow and heat transfer characters of different micro heat sinks, the thermal-enhanced factor is defined as below

(17) $$\eta =\frac{Nu/Nu_{0}}{{\left(f/f_{0}\right)}^{1/3}}$$

where *Nu*_0_ and *f*_0_ are the Nusselt number and friction factor of the reference microchannel DL-R.

## 3. Results and Discussion

### 3.1. Validation of Numerical Methods

**Figure 4:**
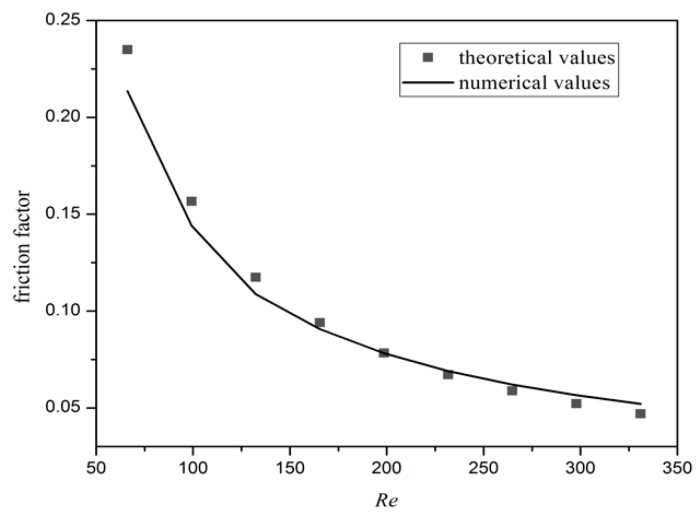
Variation of the friction factor vs. Reynolds number for DL-R micromachine structure.

**Figure 5:**
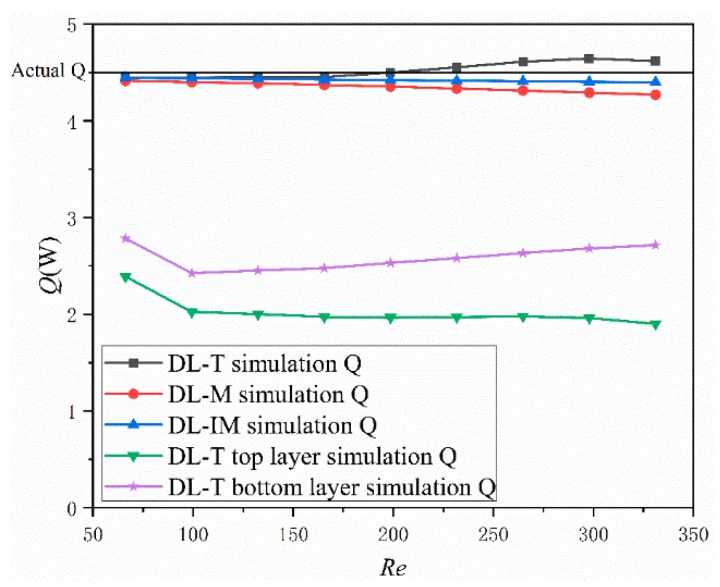
Comparison of heat input in different microchannels.

The friction factor of the DL-R structure is calculated with the above numerical method and compared with that derived from the theoretical model expressed in Equation (7) for validation purposes. As shown in Figure 4, The numerical result agrees with the theoretical data with a maximum error of 5%. The simulated value (computed by Equation (9)) of the heat input *Q* is also compared with the actual heat input as shown in Figure 5. The Q values in the top and bottom layer are calculated simultaneously in the DL-T, and the sum of them equals the total simulated Q value in DL-T. As the bottom layer is near to the heating wall, the simulated Q value at the bottom layer is higher than that at the top layer. The actual heat input is determined as the product of the heat flux (1 × 10^6^ W/m^2^) and the heat sink area (3 × 10^−3^ m × 1.5 × 10^−3^ m) and equals to 4.5 W. It is obvious that the numerical results are in excellent agreement with the actual value, with a maximum error of ±3%. Moreover, the simulated *Q* value at the bottom layer is higher than that at the top layer, which is also testified by Zhai et al. [13]. Therefore, it can be concluded that the numerical method is valid to predict the heat transfer performance of the MCHS.

### 3.2. Flow Resistance Character Analysis

**Figure 6:**
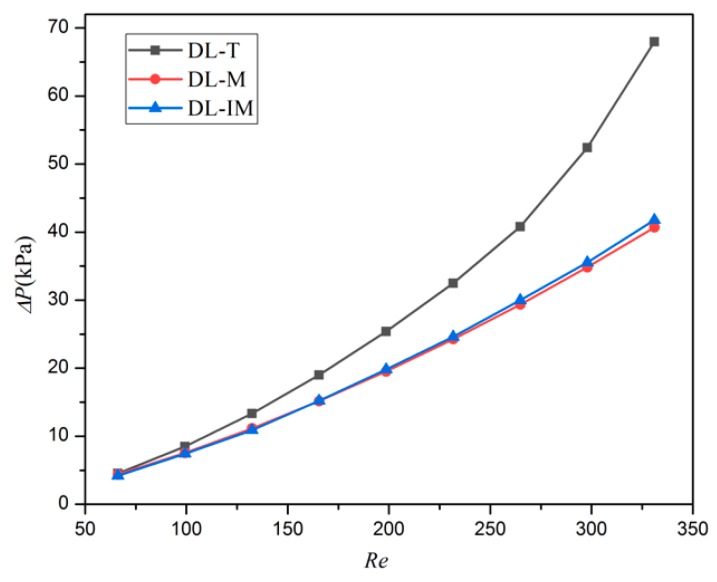
Pressure drop in DL-T, DL-M and DL-IM structure in different Reynolds number conditions.

As mentioned above, the pressure difference is one important specification for micro heat sink performance. Figure 6 illustrates the simulated results of the area weighted average pressure drop at different Reynolds number conditions for DL-T, DL-M and DL-IM. The pressure drop increases monotonously when increasing the inlet velocity (Reynolds number), and the pressure drop of DL-M and DL-IM is significantly lower than that of the DL-T. The main reason is that the flow area of DL-M and DL-IM is larger than that of DL-T. Furthermore, it can be seen that the pressure drop in DL-IM is slightly lower than that of DL-M in all the Reynolds number conditions. 

**Figure 7:**
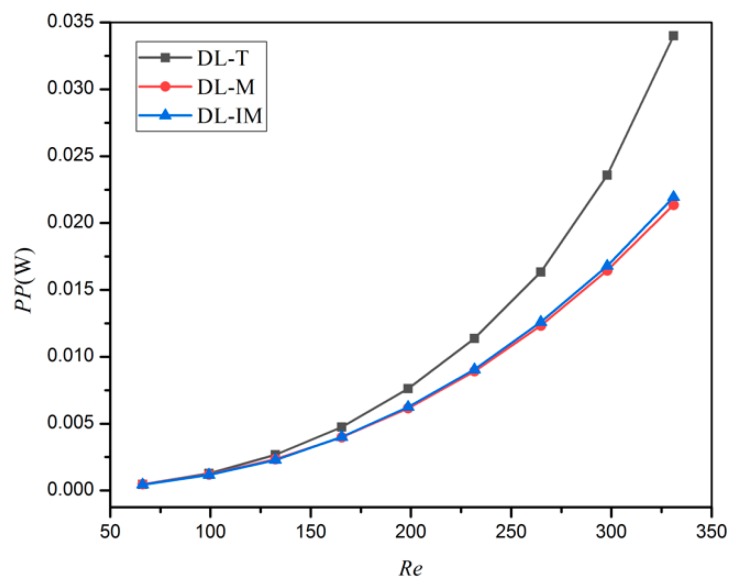
Pumping power of DL-T, DL-M and DL-IM structure in different Reynolds number conditions.

Figure 7 shows the pumping power of different micro heat sinks in different Reynolds number conditions after taking into account the flow volume rate influence. The pumping power shows a similar trend with the pressure drop. This can be explained as the DL-T has the smallest flow area and inlet section area, and is thus with the highest flow resistance in the same flow condition. DL-M and DL-IM use the slot subchannel to converge the coolant, and thus can decrease the flow resistance of the coolant. The coolants in DL-IM will mix at the subchannel intersection, so it has more flow path option and shows the lower resistance performance.

### 3.3. Heat Transfer Analysis

**Figure 8:**
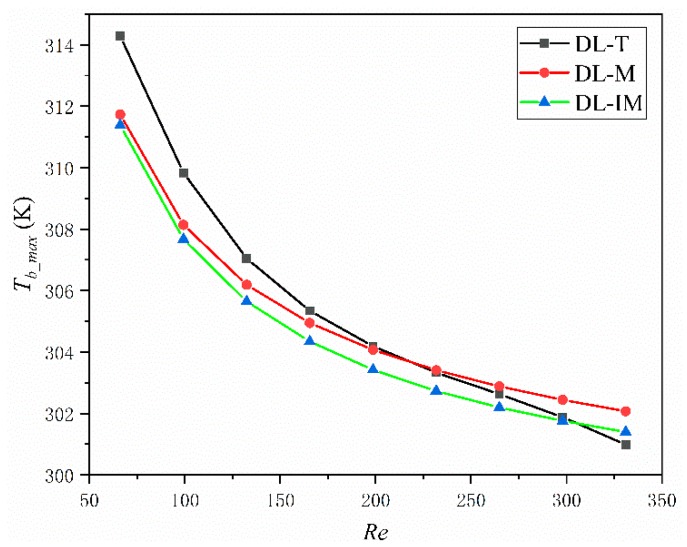
Maximum temperature on the base surface of DL-T, DL-M and DL-IM structure in different Reynolds number conditions.

**Figure 9:**
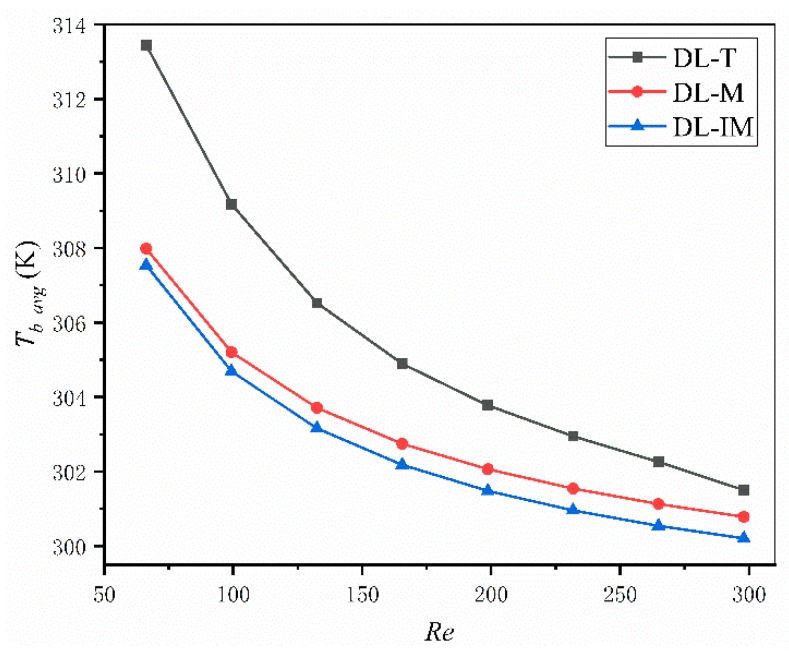
Area weighted average temperature on the base surface of DL-T, DL-M and DL-IM structure in different Reynolds number conditions.

**Figure 10:**
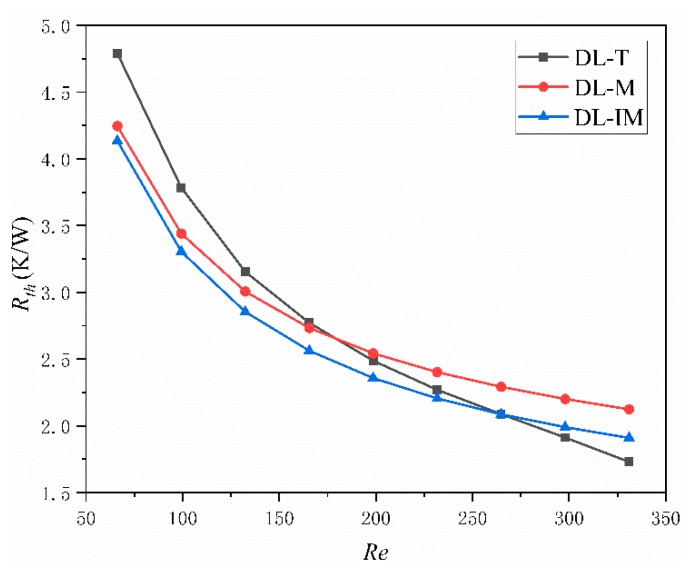
Thermal resistance of DL-T, DL-M and DL-IM in different Reynolds number conditions.

**Figure 11:**
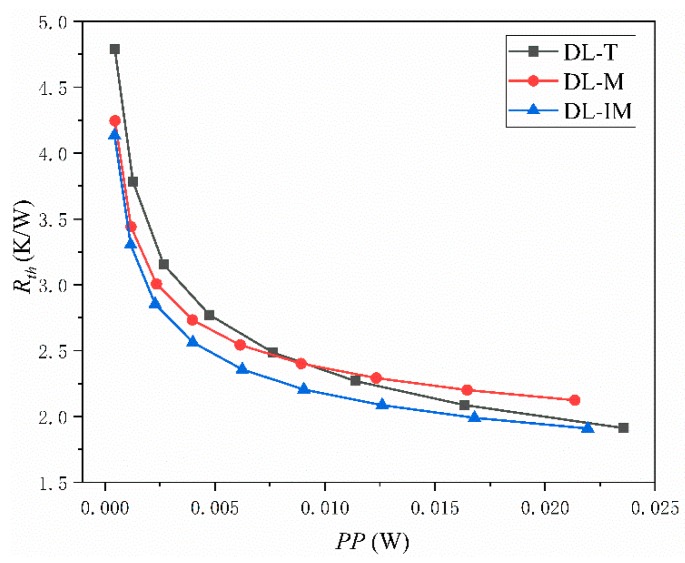
Thermal resistance of DL-T, DL-M and DL-IM at different pumping powers.

Thermal characters are the most important measurements for a micro heat sink. In an effective micro sink, a low maximum and low average temperature across the base surface and a low thermal resistance are highly desired. Figure 8, Figure 9 and Figure 10 compared these parameters in DL-T, DL-M and DL-IM in different inlet flow conditions. Figure 8 and Figure 9 show the variations of the maximum temperature and the average temperature across the base surface. It is clearly seen that the maximum and average temperature decrease with increasing Reynolds number, and DL-IM has the lowest maximum and average temperature. Figure 10 illustrates the variations of thermal resistance, showing DL-IM has the lowest thermal resistance, and DL-T has the lower thermal resistance than DL-M when Re > 180. It is also found that DL-T has the lowest thermal resistance for the highest Re number, and also as shown Figure 8, DL-T has the lowest maximum temperature on the base surface for the highest Re number. This is because for a high Re number, the fluid disturbance in DL-T increases dramatically, however, the pressure difference has been sacrificed, as shown Figure 6, which has an abrupt rise for high Re number. Nevertheless, most people only focus on lower Re number conditions of micro heat sinks. Figure 11 shows the relationship between thermal resistance and required pumping power; one can easily find that at a given pumping power, DL-IM exhibits the lowest thermal resistance and DL-T has lower thermal resistance than DL-IM when *PP* > 0.09 W, that is because DL-T has the lowest maximum temperature on the base surface for the high Re number, as shown in Figure 8. 

**Figure 12:**
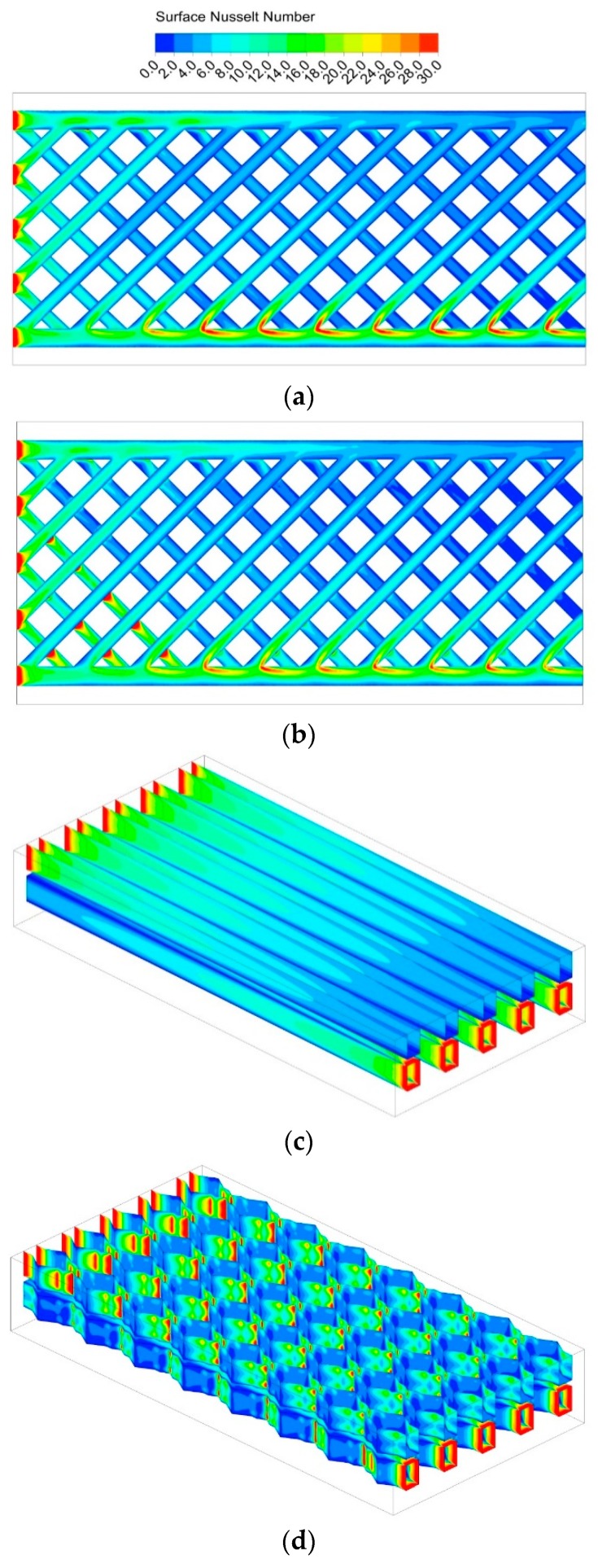
*Nu* number distribution of (**a**) DL-M channel, (**b**) DL-IM channel, (**c**) DL-R channel, and (**d**) DL-T channel when *Re* = 132.

The Nusselt number represents the enhancement of heat transfer through a fluid layer as a result of convection relative to conduction across the same fluid layer, and it can be calculated using Equation (11) so the heat flux between the coolant and solid substrate distribution can be better understood from the local Nusselt number distribution along the channel walls. Figure 12 illustrates the local Nu number contours of the four configurations at the Reynolds number of 132. For DL-R and DL-T, the surface Nusselt number along all the channel walls is shown in Figure 12c,d, the Nu values are both high at the inlet area, low around the channels corners, and decrease along the flow direction, which can reflect the entrance effect is obviously. This phenomenon and simulated values magnitude order are all in agreement with previous experimental data, as in Qu et al.’s research [27]. It can also be observed there is some fluctuation for Nu in the triangular cavity region of the DL-T structure, that is due to the flow disturbance. It can be seen that the convection heat transfer of DL-T is better than that of DL-R. 

On the other hand, the surface Nusselt number of DL-M and DL-IM on the channel bottom wall is shown in Figure 12a,b; the Nu values of DL-M and DL-M are much larger than that of the DL-R and DL-T, demonstrating a better heat transfer performance. In addition, the heat transfer performance of DL-IM is better than that of DL-M due to the coolant mixing in the subchannel intersection area of the two layers, which is verified by the bigger Nusselt number around the intersection area of DL-IM compared to that in DL-M. It is also found that the Nusselt number in the turning point (junction of slot subchannel and matrix subchannel) is significantly large, which means the heat transfer enhancement is induced around the turning point.

From the above analysis, heat flux between the coolant and solid substrate distribution can be better understood from the local Nusselt number distribution along the channel walls and we can reveal a basic principle that the coolant will has stronger heat transfer capacity where strong disturbance is produced. The coolant flow boundary layer separation and reattachment can be easily found in the conventional ribbed channels; besides the above flow characters, the coolant in DL-M or DL-IM can also produce stronger mixing and vortex.

**Figure 13:**
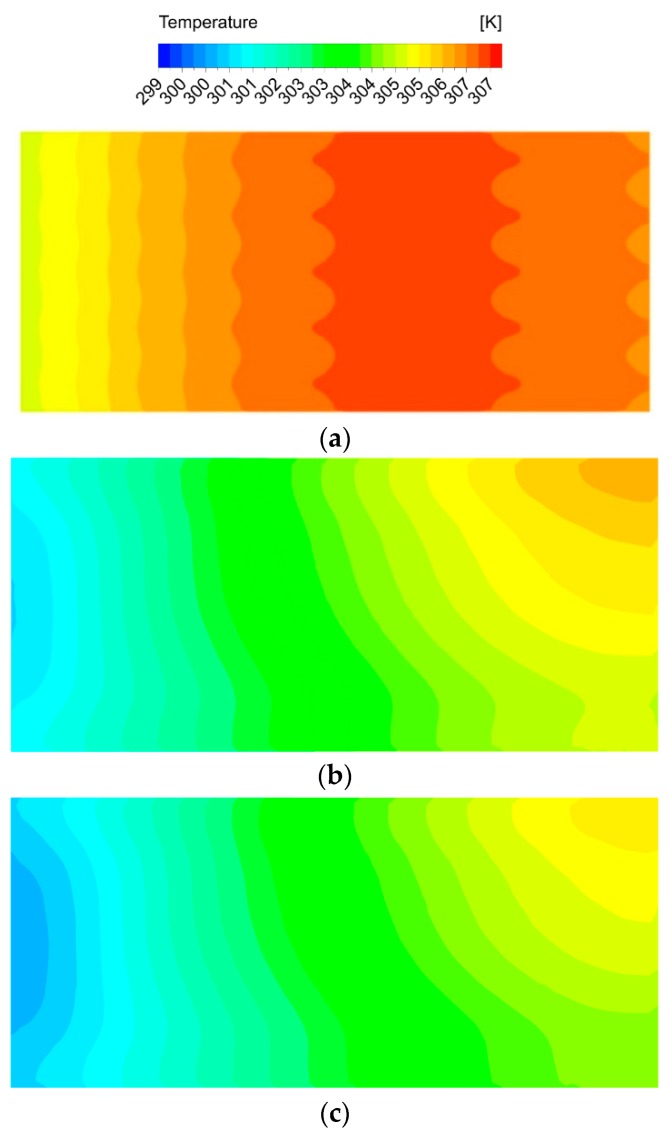
The bottom wall temperature contour of (**a**) DL-T, (**b**) DL-M and (**c**) DL-IM.

Figure 13 shows the temperature distribution at the bottom wall of DL-T, DL-M and DL-IM. The coolant flows from left to right. The average temperature level of DL-IM is much lower than that of DL-T and DL-M. As the coolant is counter-flow in DL-T, the temperature distribution of DL-T is largely different from that of DL-M or DL-IM, whose highest temperature region is on the right hand side. A zigzag distribution is also observed on the temperature line, which is induced by DL-T with triangular ribs and triangular cavities in the flow channels. Comparing Figure 13b,c, the temperature distributions of DL-M and DL-IM are similar to each other. The highest temperature region is around the top right corner, which is because DL-M and DL-IM have the matrix subchannel structure and coolant flows in parallel. These results are in agreement with previous experimental data such as in Wei et al. and Qu et al.’s researches [4,27], which also reported that the temperature distribution increased at the inlet region first and then decreased in the counter-flow condition.

**Figure 14:**
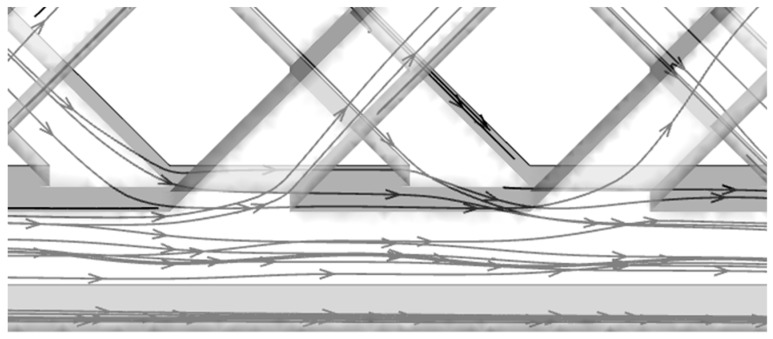
Streamlines at the intersections of subchannels and slot (Re=132).

**Figure 15:**
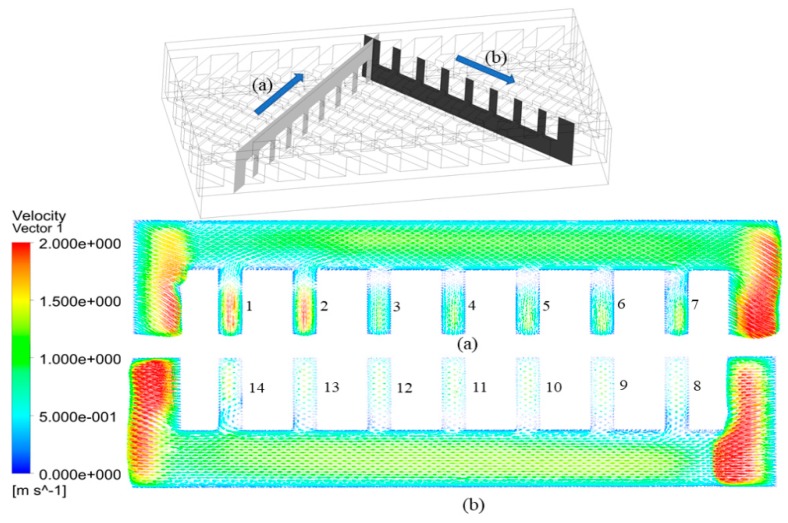
Velocity vectors on the mid-span sections of (**a**) top layer subchannel and (**b**) bottom layer subchannel in the DL-IM channel when Re = 132.

As shown in Figure 14, some coolant turns twice the rib angle and flows into the subchannels in the other layer, so the mass transfer occurs at the intersections of the subchannels and the slot. We can also find a small amount of coolant flows into the subchannel, while a large amount of coolant still flows in the slot. In the trailing-edge cooling system for gas turbine blades, the coolant will directly impinge on walls around the turning bend area, but the presence of a slot subchannel in DL-M or DL-IM can weaken the flow resistance. This flow pattern is repeated at all the intersections of slot subchannels and matrix subchannels; the final result is that mass flow increases in the slot subchannels and decreases in the matrix subchannels, so it is easy to understand why the coolant flow in the slot is unobstructed. For further analysis, the velocity vectors on the mid-span sections of a subchannel of DL-IM at Reynolds number of 132 are shown in Figure 15. The cross sections of top and bottom layer subchannels are labeled from 1 to 7 and from 8 to 14, respectively. As shown in Figure 15a, significant mass transfer between subchannels occurrs at the first two intersections, and a certain amount of coolant flows into the bulk flow from the opposite subchannel and produces a separation zone. These regions, with a pressure gradient between two layers, are occupied by low velocity coolant, and the flow is chaotic in the contact area of sections from 3 to 5. Under the effect of the adverse pressure gradient near the sidewall, a certain amount of the coolant is driven into sections 6 and 7. Figure 15b shows the flow field after turning; it is observed that the bulk flow in the downstream is accelerated compared to that in the upstream in Figure 15a due to the positive pressure gradient effect. Near the bulk flow turning, low velocity dominates in sections 8 and 9, and the coolant is pushed in the bulk flow from the opposite subchannel. Again, under the pressure gradient, the coolant flows into the opposite subchannel in the downstream sections (sections 12, 13 and 14). All the complete or incomplete vortexes are formed in the cross sections, which reveals the key role of the turning point in the formation of the dominant stream-wise vortexes.

### 3.4. Overall Performance Evaluation

**Figure 16:**
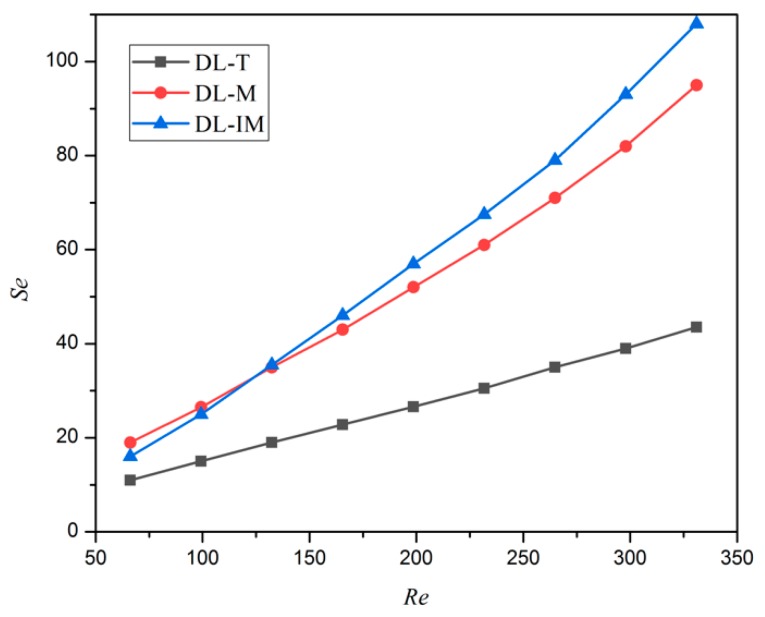
The variation of the secondary flow intensity vs. the Reynolds number.

The intensity of the secondary flow (*Se*) for DL-T, DL-M and DL-IM is compared in Figure 16. It shows that *S*e increases with *Re* in all three cases. Moreover, the secondary flow intensity of DL-M and DL-IM is much higher than that of DL-T, and the flow intensity of DL-IM is slightly higher than that of DL-M unless *Re* < 130. It implies that the configuration of the matrix and slot subchannel induces more intense flow at the cross section of DL-M and DL-IM. The coolant flows along the subchannel and changes the flow direction with twice the rib angle. Thus, the vortices are easily formed in the intersection area and slot subchannel where the strong coolant mixing is produced.

**Figure 17:**
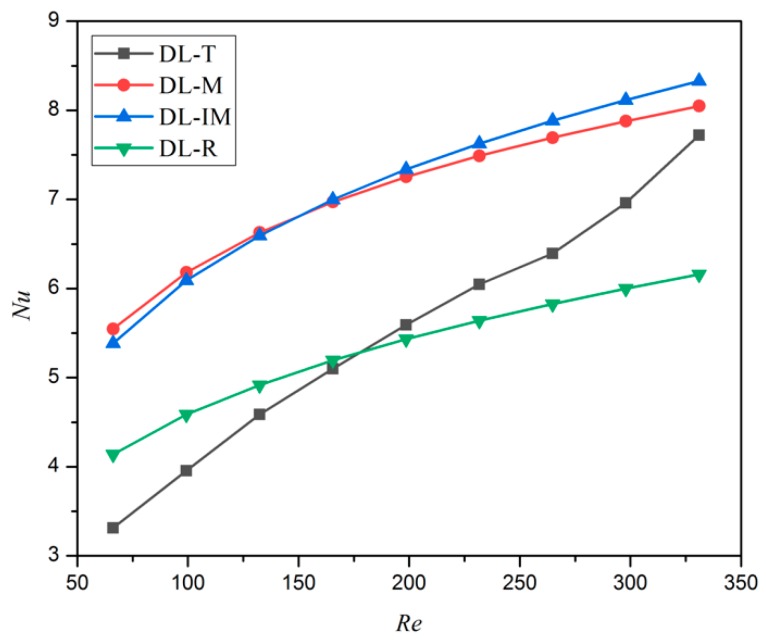
The variation of the Nusselt number vs. the Reynolds number.

Figure 17 presents the variation of the Nusselt number as a function of the Reynolds number for all four microchannels. Generally, in a higher Re condition, Nu number is larger and the flow disturbance and the heat convection are enhanced. A higher value of Nu is observed for DL-IM and DL-M, and then followed by the DL-T and DL-R. Analyzing the phenomenon, it can be concluded that the matrix structure and turning bend of DL-M and DL-IM can disturb the hydrodynamic and thermal boundary layers, thus enhancing the flow disturbance. Moreover, the coolant can fully mix in the interlinked intersection area of different layers, and thus, mass and heat transfer are enhanced. The Nusselt number vs. the Reynolds number of DL-R has a similar trend to Zhai et al.’s research [14].

**Figure 18:**
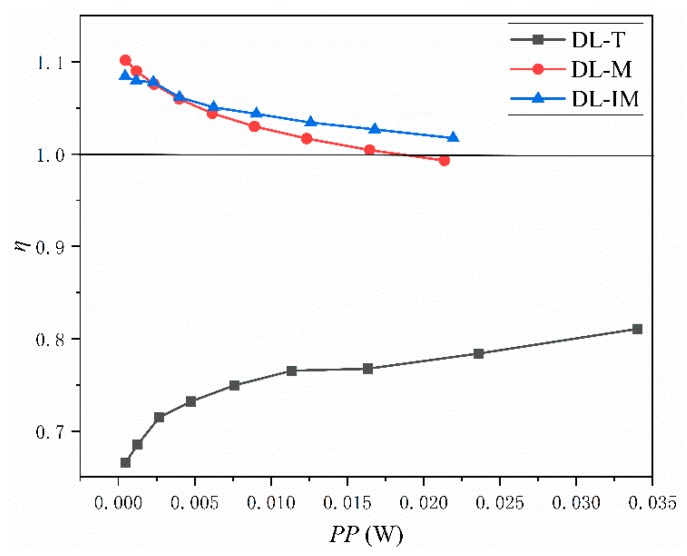
Thermal-enhanced factor vs. pumping power.

Figure 18 shows the variation of the thermal-enhanced factor along the pumping power for the three cases. As mentioned above, the simulation result of the DL-R structure under counter-flow condition is used as the reference value. It can be seen that the thermal-enhanced factors of DL-M and DL-IM are more than one in almost all pumping power conditions, and they decrease as the pumping power increases. This indicates a better performance of DL-M and DL-IM in low Re conditions. This is because with a high Re number, the flow inertia resistance will be large and the thermal enhancement factor will decrease. On the other hand, the thermal-enhanced factor of DL-T is smaller than one, and increases with the pumping power. This is highly related to the rapid increased Nu of DL-T in the high Re number condition in Figure 17. Therefore, it can be concluded that the overall performance of DL-M and DL-IM is better than that of DL-R in the counter flow condition, and the overall performance of DL-T is inferior to that of DL-R. The thermal-enhanced factor vs. pumping power of DL-T has a similar trend to Zhai et al.’s research [23,28].

## 4. Conclusions

In the past decades, numerous researches have pointed out that double-layered micro heat sinks can be effective techniques to meet the ever-increasing heat load of the future generation of electronic devices. In order to improve the cooling capacity, two new types of the double-layered matrix micro heat sinks have been presented and investigated numerically. The results have also been compared with the doubled-layered rectangular and the double-layered triangle microchannel in detail under a Reynolds number ranging from 60 to 350. Some specific conclusions can be summarized as follows:

(1) The effect of the matrix and slot subchannel arrangement on fluid flow and heat transfer is significant. The double-layered matrix micro heat sinks (especially DL-IM) has the lowest average base surface temperature and pressure drop, and also the highest Nusselt number and secondary flow intensity, which shows the best overall thermal performance.
(2) In DL-M and DL-IM, the coolant flows along the subchannel and then changes the flow direction into the other layer subchannel, in which more intense flow and stronger disturbance are induced.
(3) In DL-IM, the coolant can be fully mixed in the interlinked intersection area of different layers in the matrix subchannel, thus vortices are easily formed and mass and heat transfer are significantly enhanced.

## Figures and Tables

**Table 1:** Nomenclature used in the MCHS design and calculation.

Nomenclature			
*A_ch_*	contact area between solid and fluid (m^2^)	*Se*	intensity of the secondary flow
*c_pf_*	specific heat capacity	*u_m_*	average velocity of the coolant (m/s)
*D_h_*	hydrodynamic diameter (mm)	*W*	micro heat sinks width (m)
*f*	frication factor	*∆P*	pressure drop (Pa)
*h*	heat transfer coefficient	Greek symbols	
*H*	micro heat sinks height (mm)	*μ*	dynamic viscosity (N/m^2^·s)
*H_ch_*	top or bottom layer microchannel height (mm)	*λ*	thermal conductivity (W/(m·K))
*W_ch_*	microchannel width (mm)	*ρ*	Density (kg/m^3^)
*H’_ch_*	slot microchannel height (mm)	*ω*	Vorticity (s^−1^)
*H_b_*	height of substrate (mm)	Subscripts	
*L*	micro heat sinks length (mm)	*b*	bottom
*Nu*	Nusselt number	*f*	fluid
*pp*	pumping power (W)	*s*	solid
*Q*	heat input (W)	*in*	inlet
$\dot{m}$	mass flow rate (kg/m^3^)	*out*	outlet
$\dot{v}$	volume flow rate(m^3^/s)	*0*	reference value

**Table 2:** Dimension of micro heat sink(mm).

Microchannel	*L*	*W*	*H*	*H_ch_*_1_	*H_ch_*_2_	*H’_ch_*_2_	*H_s_*	*H_b_*	*W_ch_*	*W_b_*
DL-R	3	1.5	0.6	0.2	0.2	–	0.05	0.15	0.1	0.2
DL-T	3	1.5	0.6	0.2	0.2	–	0.05	0.15	0.1	0.2
DL-M	3	1.5	0.6	0.2	0.2	0.45	0.05	0.15	0.1	0.2
DL-IM	3	1.5	0.6	0.2	0.2	0.45	0.05	0.15	0.1	0.2
